# Combined Edge Loss UNet for Optimized Segmentation in Total Knee Arthroplasty Preoperative Planning

**DOI:** 10.3390/bioengineering10121433

**Published:** 2023-12-16

**Authors:** Luca Marsilio, Andrea Moglia, Matteo Rossi, Alfonso Manzotti, Luca Mainardi, Pietro Cerveri

**Affiliations:** 1 Department of Electronics, Information and Bioengineering, Politecnico di Milano, 20133 Milan, Italy; andrea.moglia@polimi.it (A.M.); matteo2.rossi@polimi.it (M.R.); luca.mainardi@polimi.it (L.M.); 2 Hospital ASST FBF-Sacco, 20157 Milan, Italy; alfonso.manzotti@asst-fbf-sacco.it

**Keywords:** total knee arthroplasty, orthopedic surgery, preoperative planning, artificial intelligence, automatic segmentation, UNet, CT segmentation, 3D bone reconstruction, clinical translation

## Abstract

Bone segmentation and 3D reconstruction are crucial for total knee arthroplasty (TKA) surgical planning with Personalized Surgical Instruments (PSIs). Traditional semi-automatic approaches are time-consuming and operator-dependent, although they provide reliable outcomes. Moreover, the recent expansion of artificial intelligence (AI) tools towards various medical domains is transforming modern healthcare. Accordingly, this study introduces an automated AI-based pipeline to replace the current operator-based tibia and femur 3D reconstruction procedure enhancing TKA preoperative planning. Leveraging an 822 CT image dataset, a novel patch-based method and an improved segmentation label generation algorithm were coupled to a Combined Edge Loss UNet (CEL-UNet), a novel CNN architecture featuring an additional decoding branch to boost the bone boundary segmentation. Root Mean Squared Errors and Hausdorff distances compared the predicted surfaces to the reference bones showing median and interquartile values of 0.26 (0.19–0.36) mm and 0.24 (0.18–0.32) mm, and of 1.06 (0.73–2.15) mm and 1.43 (0.82–2.86) mm for the tibia and femur, respectively, outperforming previous results of our group, state-of-the-art, and UNet models. A feasibility analysis for a PSI-based surgical plan revealed sub-millimetric distance errors and sub-angular alignment uncertainties in the PSI contact areas and the two cutting planes. Finally, operational environment testing underscored the pipeline’s efficiency. More than half of the processed cases complied with the PSI prototyping requirements, reducing the overall time from 35 min to 13.1 s, while the remaining ones underwent a manual refinement step to achieve such PSI requirements, performing the procedure four to eleven times faster than the manufacturer standards. To conclude, this research advocates the need for real-world applicability and optimization of AI solutions in orthopedic surgical practice.

## 1. Introduction

Total knee arthroplasty (TKA) is a surgical procedure designed to address advanced degenerative knee diseases, such as osteoarthritis, rheumatoid arthritis, or post-traumatic arthritis by replacing or reconstructing the joint [1]. Considering the steady increase in the elderly population, primary and therefore revision TKAs are expected to increase over the next decades [2,3,4]. Since an accurate alignment in primary TKAs is related to a reduced need for revision procedures, enhancing the accuracy of TKA techniques is essential [3]. Accordingly, recent attention was given to Personalized Surgery Instruments (PSIs), whose deployment resulted in a more repeatable neutral postoperative alignment and reduced surgical time, with no further intra-operative complications [5,6,7]. PSIs, designed for TKA intervention, are patient-specific cutting jigs replicating the contours of a patient’s distal femur and proximal tibia. They enable surgeons to execute precise bone cuts, aligning the knee implant according to computer-based planning for optimal placement [8]. PSI manufacture and deployment require the availability of the digital three-dimensional (3D) surfaces of the tibia and femur [9,10]. These volumes are needed to produce the disposable instrumentation, assess the ideal femoral and tibial resection planes, select the prosthesis dimensions, and optimize its placement to reduce the risk of inadequate fitting and related loosening of the implants [11]. Bony surfaces are obtained from the segmentation and 3D reconstruction of preoperative volumetric imaging, including Computed Tomography (CT) and Magnetic Resonance Imaging (MRI). However, the variability in bone shape and dimensions, the presence of severe pathological conditions narrowing the intra-articular spaces, and the development of osteophytes, result in highly irregular profiles, making the delineation of surface boundaries challenging even for expert radiologists [12,13]. Furthermore, achieving sub-millimetric alignment between the patient’s bone surface and the jig footprint is a requirement for a successful knee surgery guided by PSIs [14,15]. Therefore, tibia and femur 3D reconstructions are of paramount importance in ensuring accurate matching of the instrumentation to the patient’s anatomy, and consequently, the reliability of the entire surgical planning. To meet the strict accuracy requirements, bone segmentation is still performed by expert radiologists supported by clinical image management and visualization software such as Mimics, version 2.0.99.7 (Materialise NV, Leuven, Belgium) and 3D Slicer, version 5.2.2 (by Slicer Community) [11]. Semi-automated approaches provide more control over the segmentation process, enabling a visual inspection and correction of critical areas. Nevertheless, dealing with complex anatomical structures is time-consuming and the quality of segmentation may vary between operators, introducing interobserver variability [16,17]. Recent years have seen the rise in artificial intelligence (AI) algorithms, transforming modern patient care across various branches of medicine [18,19,20]. Specifically, Convolutional Neural Networks (CNNs) play a pivotal role in the realm of AI, being devoted to image analysis and automated segmentation. They learn specific information patterns at different resolutions, enhancing the network’s ability to extract both localized and contextual information. Their performances have been tested in various medical areas with different imaging equipment. However, most studies have lacked translational analysis towards the clinical world, posing a limit to the application of these innovative technologies in the healthcare domain. In this work, we proposed a pipeline procedure based on a multi-task CNN devoted to bone segmentation in CT scans. The network was trained on a large dataset, and its performances were tested in an operational TKA environment (Technology Readiness Level, TRL-5).

### 1.1. State of the Art

Following the introduction of the UNet [21], a symmetric encoding–decoding CNN, and its derived architectural variations, including the nnUNet [22], biomedical image segmentation experienced an unprecedented push forward, with these networks being scalable to a wide range of clinical applications, including orthopedics [23,24]. Fully automated algorithms built upon CNNs can rapidly process large input images offering consistency in segmentation results. However, since each outcome completely relies on the network output, 3D reconstruction errors larger than 1 mm might lead to the withdrawal of the PSI technique in favor of the traditional more invasive surgery [14]. In recent years, several studies have addressed bone segmentation tasks enhancing state-of-the-art performances through architectural or methodological innovations. Two novel data augmentation methods were introduced in [25] to overcome the limited data availability, boosting the overall results. In [26] different segmentation performances were compared by training a standard UNet architecture following three different pipelines. A new architectural variant was introduced in [27], where an automatic segmentation algorithm based on the VNet-C network was examined. In [28], a pure dilated residual UNet was proposed for the tibia and femur segmentation employing dilated convolution to increase the receptive field. Nonetheless, the impacts of osteophyte formation and bone deformation on local segmentation errors were not addressed in these studies, underestimating their impact on surgical planning. As a result, the clinical translation of these solutions was significantly hindered. In [29], our group presented a 3D-UNet architecture adapted to knee bone segmentation in CT images, with the clinical aim of preoperative planning in TKA surgery based on PSI. In [30], we proposed a novel multi-task UNet architecture, called CEL-UNet, tailored to tackle bone segmentation uncertainties in very irregular shapes and large deformities. CEL-UNet outperformed the benchmark networks, setting the basis for it to be integrated into an automated preoperative pipeline. However, both prior studies leveraged downsampled CT scans to fit GPU memory requirements, reducing both input and output resolution. For this reason, a particular focus should also be provided to non-architectural factors such as image preprocessing and label generation since they play a substantial role in performance improvement [31]. Dealing with large input images, such as CT and MRI, might preclude network training with less powerful hardware. Downsampling the original voxel size reduces volume dimensions and guarantees training while sacrificing resolution and output accuracy. Alternatively, patch-based methods provide patches of the input image at the original resolution. Larger patches give more contextual information but lead to smaller batch sizes, which increase the variance in stochastic gradient and decrease optimization [32]. Finally, bone boundary annotations must be consistent with the anatomy contours, otherwise, redundant inaccuracies might bias the segmentation model towards predicting the same uncertainties on new data [33].

### 1.2. Work Contribution

The advancements proposed in this work address both methodological and translational aspects, aiming to integrate a robust and reliable AI-based tool into a TKA preoperative planning workflow tackling the time-consuming and labor-intensive operations of manual segmentation. The main contributions are: (1) the assessment of how tailored handling of non-architectural factors, including a patch-based method and an improved segmentation label generation algorithm, could affect segmentation and 3D reconstruction outcomes when dealing with highly irregular boundary profiles; (2) a quantitative morphological matching quality analysis between the reconstructed anatomy and the PSIs on true contact areas; (3) a quality evaluation test of the reconstructed surfaces in a real TKA preoperative operational environment to assess the clinical translation potential of our project; and (4) a time evaluation for each step of the proposed automated pipeline, highlighting the drastic time reduction with this innovative approach compared to the traditional manual bone reconstruction.

This paper is structured as follows: In Section 2, we describe the dataset, training set preparation, network architecture, and test design. In Section 3, we report the results, while in Section 4, we discuss the main findings, compare them with published literature, and present the technical challenges and work limitations. The conclusions are reported in Section 5.

## 2. Materials and Methods

### 2.1. Dataset Description

A dataset of 876 axial CT images was provided by MEDACTA International SA (Castel San Pietro, Switzerland) in Digital Imaging and Communications in Medicine (DICOM) standard format, acquired in the context of preoperative planning for TKA intervention from 2017 to 2022. Upon diagnosis, patients indicated localized knee pain and instability. Clinical observations revealed varying degrees of cartilage defects, femoral osteophytes, and shape abnormalities primarily located in the tibial plateau and condylar regions of the distal femur. Out of the 876 cases, 54 were excluded due to inner metal parts, namely screws, implants, and plaques, with their segmentation being out of the scope of this work. The remaining 822 images (397 males and 425 females, 409 right against 413 left knees) were considered in the present study. CT scans were acquired with various imaging devices, mostly at 512 × 512 pixels and 430 slices on average, with variable voxel size, ranging from 0.24 to 0.94 mm, and axial slicing from 0.3 to 1.0 mm. Alongside CT images, proximal tibia and distal femur corresponding reconstructed surfaces were provided in STL format. Reference bony surfaces were manually generated by expert radiological operators from semi-automatic segmentation performed using Mimics software, version 2.0.99.7. These STL data were exploited to produce the segmentation labels for the training procedure and to assess both segmentation and volume reconstruction performances. For this study, 75% of the dataset, corresponding to 622 cases (301 males and 321 females, 312 right against 310 left) were randomly selected for training, while the remaining 25%, 200 images, defined the test set. This split percentage allowed for a broader evaluation of the model’s performance on unseen data, enhancing the reliability of the findings.

### 2.2. Training Set Preparation

**Figure 1:**
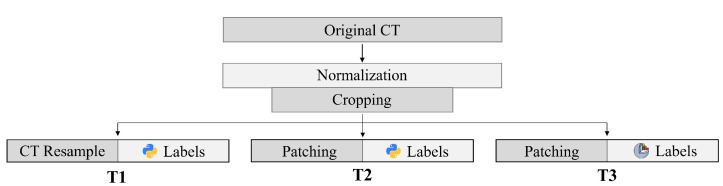
Training sets generation summary: normalization and cropping were applied to all CTs. *T1* is generated by resampling the cropped volumes to 192 × 192 × 192 and with custom Python-generated segmentation labels. For *T2*, the same labels were produced, while CTs were patched to 160 × 160 × 160. Finally, *T3* features patched volumes and segmentation labels obtained through the 3D Slicer procedure.

Three separate training sets (T1, T2, T3) were generated from the same original dataset, as summarized in Figure 1. Different data preparation and label generation strategies were implemented to determine whether non-architectural factors might play a substantial role in performance improvement [31]. Since CT scans originated from different scanning machinery, a voxel intensity normalization was applied to produce a consistent distribution. First, each voxel intensity value was scaled to the Hounsfield Unit (HU) range [34] according to the *rescale slope* and *rescale intercept* parameters gathered in the DICOM header. Voxel values were then clipped between -1024, HU notation for air, and 2500, high-density cortical bone [35]. Finally, intensities were shifted to positive numbers (from 0 to 3524) and normalized between 0 and 1, providing a consistent distribution. Original scans were automatically cropped in the axial, coronal, and sagittal view, removing all the slices where reference segmentation of the proximal tibia and distal femur was not available, to reduce the computational overhead. T1 was generated following the description presented in [30]. Therefore, all the cropped volumes were resampled to a fixed dimension of 192 × 192 × 192, reducing the volume size to cope with hardware limitations, at the cost of losing voxel resolution. For T2, a patch-based method was implemented. Each cropped CT was patched into a different number of 160 × 160 × 160 sub-volumes, depending on their initial dimension, preserving the original voxel resolution while ensuring the training procedure.

**Figure 2:**
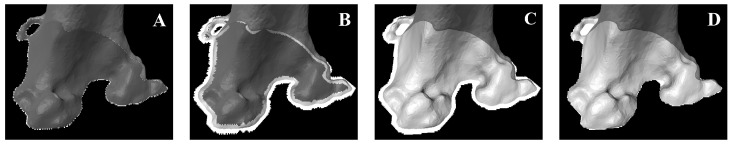
Python label generation algorithm sequence. (**A**): Intersection points between 3D reference femur and orthogonal plane; (**B**): dilation operation to make all points contiguous; (**C**): closing and filling to fill the polygonal; (**D**): erosion operation to shrink label boundaries.

Patch size was heuristically determined as a trade-off between the contextual information represented in a single patch and the resulting training batch size. Patch overlapping allowed the information loss on the CT boundaries resulting from the mismatch between CT and patch size to be reduced. The total number of training patches was 3686. For both T1 and T2, the generation of segmentation labels from the reference surfaces followed a custom automated algorithm written in Python (version 3.9.16) leveraging on the *trimesh* and *scipy* libraries, consistent with the procedure applied in [29,30], and summed up in Figure 2. The intersection points between the hollow 3D volume and the corresponding CT were computed for each axial slice, saved in a binary mask, and stacked (A). A *fill holes* method, to fill the closed perimeter, was applied to each slice of the generated binary volume where a contiguity condition was met by all the identified points. Otherwise, consecutive morphological operations, including dilation (B), closing (C), and erosion (D) were applied to comply with the previous requirement. Nevertheless, the application of these morphological operations might smooth and enlarge the original bone boundary perimeter, particularly where the intra-articular spaces are narrow, biasing a trained model to oversegment these critical areas.

**Figure 3:**
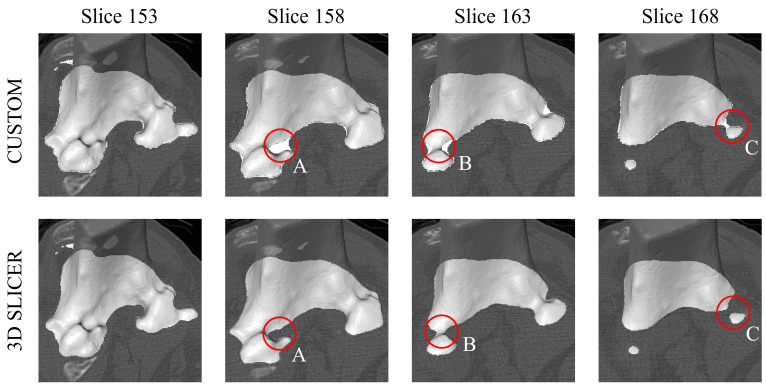
Label generation comparison. (**First row**): Custom algorithm; (**Second row**): 3D Slicer-based algorithm. For each slice of the CT, its corresponding bone reference surface and the segmentation labels (in white over the CT) are shown. A, B, and C circled areas highlight major differences in critical regions for the two approaches.

Consequently, T3 was generated with the same patch-based method as T2, while the segmentation label algorithm was modified. This time, a different automated approach was implemented in a medical image processing and visualization software: 3D Slicer. CT scans and the corresponding reference surfaces were loaded in the software as VolumeNode and LabelMapVolumeNode data, respectively. Using a sequence of built-in 3D Slicer functions, including *ExportVisibleSegmentsToLabelmapNode*, to map the 3D volumes of the tibia and femur into a binary segmentation volume, and *arrayFromVolume*, to convert it to a NumPy array, a data structure handled by our custom Python algorithm, the volume segmentation map was generated, mapped to the original CT resolution, and exported in the Nifti (*.nii*) format. Following this pipeline, labels trace the exact bone boundaries, even close to thin and narrow joint spaces, without applying morphological operators; therefore, there was no distortion to the reference surfaces while producing segmentation masks. The outcome comparison between the two algorithms is shown in Figure 3, where four CT slices, the corresponding femur reference surface, and the generated labels are shown. Circled areas A, B, and C point out some critical regions where implementing the Python algorithm resulted in superabundant labels near the narrowness of the lateral and medial femoral condyles.

### 2.3. Network Trainings and Architectures

T1, T2, and T3 were used to train a CEL-UNet architecture, built as described in [30]. This network embeds a UNet-like [21] encoder to extract features at decreasing spatial resolutions ending with the so-called bottleneck. Its peculiarity lies in the decoding path, which is split into two parallel branches, one dedicated to semantic segmentation and the other tackling bone boundary identification. The edge information acquired from this branch is aggregated to the feature maps of the main one through vertical unidirectional skip connections allowing the robustness of narrow border detection to be increased. The dataset showing the best performances with the CEL-UNet was also used to train a state-of-the-art UNet following the best architecture setup found by [29]. Two trainings were performed deploying different loss functions, namely, Distance Cross-Entropy (DCE) and Focal (FOC) loss. All training procedures and predictions were performed on a 32-core CPU and NVIDIA A100-PCIE GPU with 40 GB RAM.

### 2.4. Segmentation and 3D Reconstruction Result Analysis

Tibia and femur segmentation quality assessment was carried out by computing precision and recall, responsive for both over- and undersegmentation errors, respectively. Additionally, a measurement of intersection over union was performed by extracting Dice and Jaccard indexes. The 3D surfaces of each segmented volume were automatically built exploiting a custom algorithm based on marching cubes [9]. Reconstruction accuracy was evaluated in terms of Hausdorff distance and Root Mean Squared Error (RMSE), considering the maximum and average distance between the predicted and reference surfaces. Time evaluation performances were carried out for each step of the automated pipeline, including preprocessing, segmentation, postprocessing, and 3D reconstruction of the tibia and femur, to define the median and interquartile range (IQR) times to achieve the overall task on the same hardware exploited for the training process. Statistical tests were run deploying the non-parametric Kruskal–Wallis technique, including the Tukey–Kramer post-hoc comparison. A *p*-value less than 0.05 was considered statistically significant.

### 2.5. Quantification of PSI-Based Surgical Planning Feasibility

**Figure 4:**
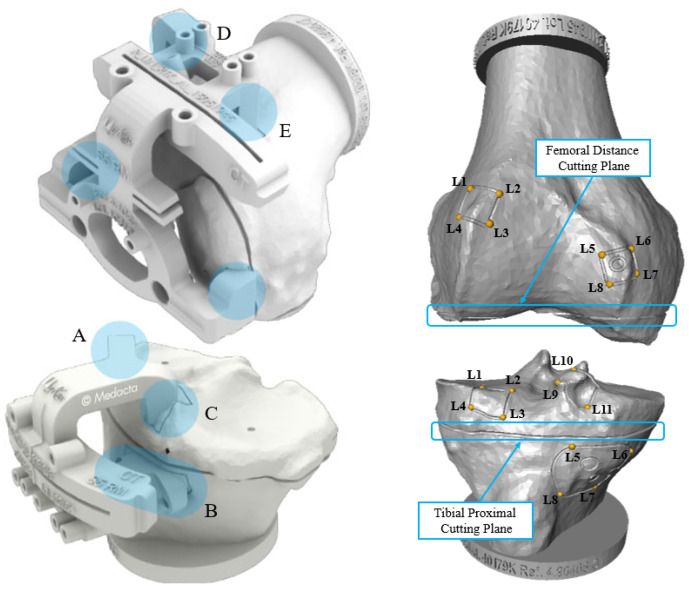
(**Left**): Femoral and tibial PSI of MyKnee system (courtesy of MEDACTA International SA) with the contact areas on the tibia (A–C) and on the femur (D,E) highlighted in light blue. (**Right**): Planning surfaces highlighting femoral distance and tibial proximal cutting planes, in light blue, and the landmark points (L1–L8 for femur and L1–L11 for tibia) defining the contact areas.

Tibia and femur automated reconstruction quality were also quantified with regard to the clinical impact on the TKA surgical planning built upon the MyKnee technology developed by MEDACTA International SA. This quantification was performed over 20 surgical cases, randomly extracted from the test set, and processed with the CEL-UNet architecture trained with the dataset that showed the best segmentation and 3D reconstruction performances. The feasibility of the PSI-based surgical planning was assessed by matching the predicted bones to their corresponding planning surfaces provided by the company. Distance errors between the PSI contact areas on the reference and on the reconstructed volumes as well as angular alignment errors of the distal femoral and proximal tibial cutting planes were computed. Specifically, three contact areas were considered for the tibia, on its medial and lateral condylar regions (A, C), and on the frontal area next to the tuberosity (B), while two regions (D, E) were considered in the frontal distal femur (Figure 4). Each one was defined on the planning surfaces by selecting either three or four landmark points at its vertices. Contact area A, for instance, is defined by landmarks L1, L2, L3, and L4. Every landmark was translated into the reconstructed volume by minimal distance criteria, and spatial distances between the reference and predicted landmarks were computed to assess the errors for each area. Furthermore, the angular alignment errors for the proximal tibia and distal femur cutting planes were computed. This time, four landmarks were picked on each planning volume following the resection sulcus, two frontally and two posteriorly. The normal direction of the plane fitting to the points was calculated for both reconstructed and planning volumes. Their angular deviation was projected on the sagittal and frontal anatomical planes, obtaining two clinically relevant measures [8,36,37].

### 2.6. Test in Operational Environment

The automated segmentation and 3D bone reconstruction pipeline performances were also assessed in the *My Knee* department of MEDACTA, committed to the production of bone surfaces from preoperative CT scans complying with their internal protocol requirements. Three operators examined a set of 30 different tibia and femur 3D models to assess their reconstruction quality. However, since they could have been biased by knowing that volumes were generated by AI-based algorithms, only 15 out of the 30 were produced through the automated pipeline from the test set data. The remaining were manually obtained by the consolidated MyKnee internal process. During the test, operators had to open the tibia and femur volumes on Mimics software, alongside the corresponding CT scan, to assess whether the reconstruction quality met the standards for the PSI guide’ production by analyzing the generated colormaps. If not, they had to manually refine the provided volume to match such requirements, and quantify the time needed to perform this operation and the required amount of time to perform the segmentation starting from scratches with their internal pipeline. The prior hypothesis is that all the manually reconstructed volumes will match the restrictive standards. This protocol was carried out in collaboration with the *quality* department of MEDACTA.

## 3. Results

### 3.1. Impact of Non-Architectural Factors on the CEL-UNet

**Figure 5:**
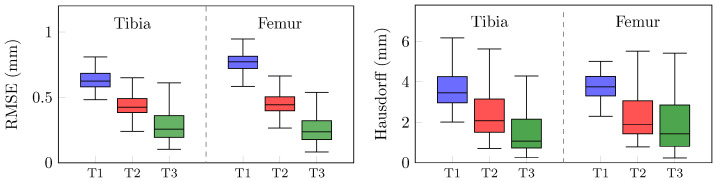
Tibia and femur 3D reconstruction error comparison in terms of RMSE (**left**) and Hausdorff distance (**right**). Boxes depict the outcomes of a CEL-UNet architecture alternatively trained with datasets T1 (blue), T2 (red), and T3 (green).

The analysis of how different non-architectural factors can impact the same CNN’s prediction ability is shown in Figure 5. It compares the 3D reconstruction errors between reference and predicted bone surfaces rather than semantic segmentation scores since segmentation labels were different across the three datasets. Statistically significant differences (*p* < 0.05) were assessed for both RMSEs and Hausdorff distances. The results, expressed in terms of the median and interquartile range (IQR), show how discrepancies fall from 0.62 (0.58–0.68) mm and 0.77 (0.72–0.81) mm for T1 for the tibia and femur, to 0.42 (0.38–0.49) mm and 0.45 (0.31–0.63) mm for T2, showing how the resolution lost due to the CT fixed resizing applied in [30] strongly affects the output accuracy. Additional improvements are registered from T2 and T3, where the RMSE dropped to 0.26 (0.19–0.36) mm and 0.24 (0.18–0.32) mm for the tibia and femur, respectively. These outcomes show how a careful label generation, accurately defining the narrow bone boundaries and thin joint spaces, directly transfers this knowledge to the network during training, bringing down both average and maximum reconstruction errors. Accordingly, the Hausdorff distance more than halved with T3, reaching 1.06 (0.73–2.15) mm for the tibia and 1.43 (0.82–2.86) mm for the femur, while T1 and T2 had distances of 3.46 (2.97–4.26) mm and 2.07 (1.50–3.77) mm for the tibia, and 3.75 (3.31–4.27) mm and 1.90 (1.43–3.06) mm for the femur, respectively.

### 3.2. CNN Architecture Comparison for Segmentation and 3D Reconstruction

**Figure 6:**
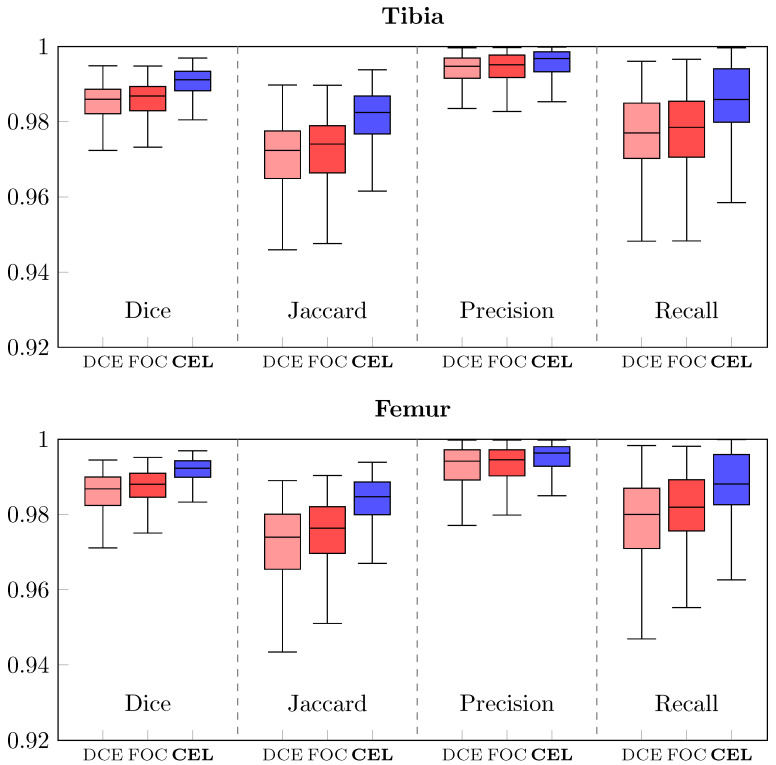
Boxplots of Dice, Jaccard, Recall, and Precision for tibia (**above**) and femur (**below**). Blue boxes depict the CEL-UNet results, while light red and dark red boxes show the UNet architecture outcomes after training with Distance Cross-Entropy (DCE) and Focal (FOC) loss, respectively.

**Figure 7:**
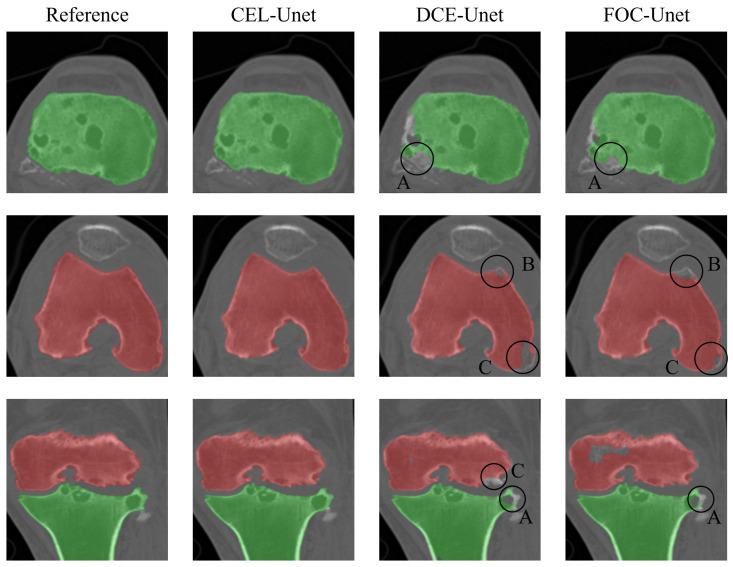
Qualitative comparison of the tibia (green) and femur (red) segmentation for case code 0782 against the reference label. CEL-UNet outputs are shown in the second column, while DCE- and FOC-UNet segmentations are in the third and fourth columns, respectively. Circled areas display segmentation errors of the UNet models in both tibial lateral (A) and femoral medial (B, C) condyles.

The results of the CEL-UNet architecture were compared to a 3D UNet, configured as in [29], alternatively compiled with two different loss functions, Distance Cross-Entropy (DCE-UNet) and Focal loss (FOC-UNet). All three networks were trained with dataset T3 since it proved its superiority against the others. Segmentation scores for the tibia and femur are plotted in Figure 6, while median and IQR values are reported in Table 1. Statistically significant differences were found between CEL-UNet and the two UNet networks for each metric computed, while *p* > 0.05 was assessed among the DCE- and FOC-UNet, meaning that the deployment of two different loss functions did not statistically change the output metric distribution. A qualitative comparison of the tibia (green) and femur (red) segmentation predictions between the three networks is shown in Figure 7 for case code 0782, belonging to the test set. The network outputs are compared to the reference label. Two axial slices showing tibia and femur cross-sections (first and second row, respectively) and one coronal slice (third row) are presented. These images display how the CEL-UNet architecture, tailored to tackle bone boundary segmentation even with damaged anatomies and narrow joint spaces, achieves its goal, while standard UNet networks fail at segmenting areas in both tibial lateral (A) and femoral medial (B, C) condyles.

**Figure 8:**
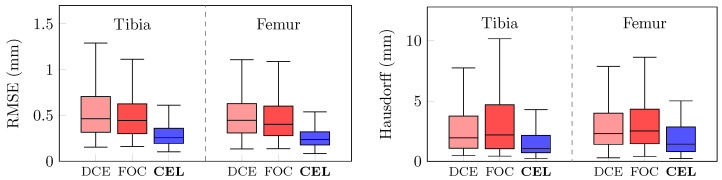
Tibia and femur 3D reconstruction error comparison in terms of RMSE (**left**) and Hausdorff distance (**right**). Blue boxes depict the CEL-UNet architecture metric distributions, while light and dark red boxes display DCE- and FOC-UNet scores, respectively.

**Figure 9:**
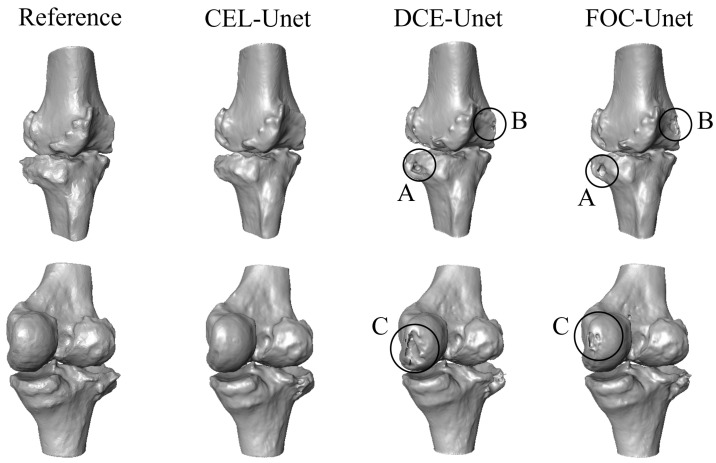
Qualitative 3D predicted reconstruction of the tibia and femur for case code 0782 against the reference label, in anterior (**above**) and posterior (**below**) view. CEL-UNet outputs are shown in the second column, while DCE- and FOC-UNet segmentations are in the third and fourth columns, respectively. Circled areas display reconstruction errors of the UNet models in both tibial lateral (A) and femoral medial (B, C) condyles.

Figure 8 compares the 3D reconstruction errors for the whole test set. Again, *p* < 0.05 was assessed for both RMSE and Hausdorff distance proving the CEL-UNet advantage against the two UNet models. Median and IQR values for the RMSE were 0.46 (0.32–0.71) mm and 0.45 (0.31–0.63) mm with DCE-UNet, while they were 0.45 (0.31–0.63) mm and 0.40 (0.28–0.60) mm with FOC-UNet, for the tibia and femur, respectively. The Hausdorff score was 1.95 (1.09–3.76) mm and 2.30 (1.41–3.99) mm with DCE-UNet, and 2.19 (1.06–4.70) mm and 2.52 (1.47–4.33) mm with FOC-UNet. A visual inspection of the tibia and femur 3D surface reconstruction for case code 0782 is illustrated in Figure 9. The segmentation errors (A, B, and C) depicted in Figure 7 for the DCE- and FOC-UNet are now visible in the meshes.

Table 2 shows the time spent to complete each operation in the inference prediction pipeline for a CEL-UNet network trained with dataset T3. CT segmentation is the longest step, with a median time of 4.4 s, while proximal tibia 3D reconstruction is generally quicker than that for the distal femur, 1.8 (1.5–2.2) s against 2.5 (2.1–2.95) s, because of the smaller dimensions of the bone portion. An overall median time of 13.1 s proves how this automated pipeline couples sub-millimetric surface reconstruction errors with a high-speed throughput.

### 3.3. Quantification of PSI-Based Surgical Planning Feasibility

**Figure 10:**
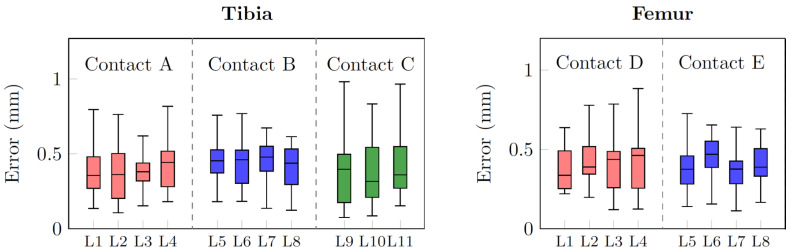
Boxplots of the error distance distributions between the landmarks picked on the reference surface and the ones identified on the predicted volume. The boxplots sharing the same color refer to the landmark points of a single contact area.(**Left**): Tibia contact areas A, B, and C. (**Right**): Femur contact areas D and E.

The error distance distributions between the landmarks picked on the reference surface and the ones identified on the predicted volume are shown in Figure 10. The group of landmarks defining the same contact area share the same color (e.g., boxes for L1, L2, L3, and L4 points defining tibial contact area A are light red). Even though tibial condylar regions (A and C on Figure 4) tend to suffer wider pathological deformations making the segmentation more challenging, all median error values reported for both the tibia and femur were below 0.5 mm, ranging between 0.31 and 0.48 mm. Such outcomes enhance the robustness of the whole pipeline by assessing the algorithm performances in local areas crucial for clinical surgery. Finally, the median and IQR ranges of the angular alignment errors projected on the frontal and sagittal planes for both the tibia and femur are indicated in Table 3.

### 3.4. Test in Operational Environment

**Figure 11:**
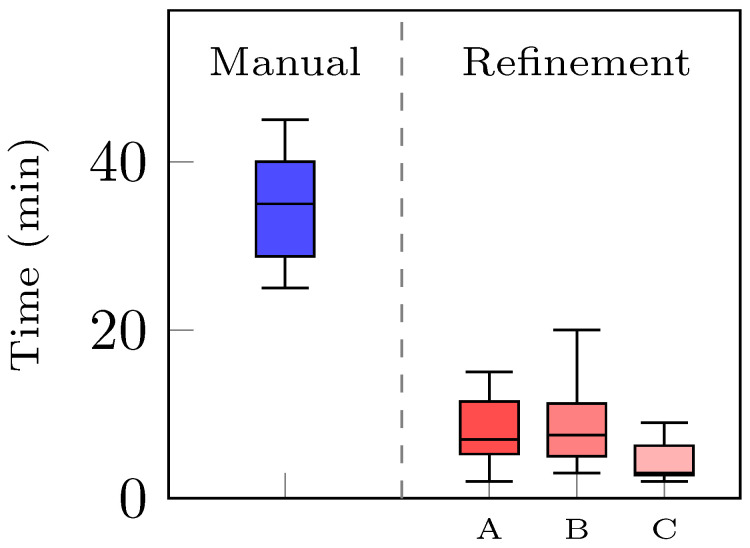
Comparison between the time needed to generate the 3D volumes of tibia and femur starting from scratches with a manual approach (blue) and the refinement time spent by three different operators, A, B, and C (red), to fit the automatically reconstructed model to the company’s requirements.

MEDACTA MyKnee operators analyzed 30 3D reconstructed volumes to assess whether they were compliant with the company standard requirements for the PSI guide production. Among them, all 15 manually generated surfaces were acceptable, confirming the prior hypothesis. For the 15 remaining ones, outputs of the AI-enhanced pipeline, the operators evenly established the same eight out of 15 tibia and femur models as compliant. The leftover reconstructed models underwent a refinement process on Mimics. The interoperator time variability to complete the procedure with the same volumes is illustrated in Figure 11. The highest value registered was 7.5 min (5–11.25) from operator B, while the lowest was 3 min (2.75–6.25) from operator C, four to 11 times faster than the actual company’s internal operations performing a manual segmentation of the same surgical cases from scratches, which is 35 min (28.8–40). Statistically significant differences were observed between operator C and the others (*p* < 0.05), revealing how the manual operation time depends on the operator’s ability.

## 4. Discussion

### 4.1. Main Findings

Automatic CT bone segmentation poses fewer obstacles with respect to the segmentation of other anatomical regions. Bones are dense structures with a high density and sharp edges, making them easier to identify compared to other soft tissues and organs. However, several pathological conditions impact their mineral density, causing bone deformations, osteophytes development, and cartilage damage, raising the segmentation complexity and driving automated algorithms to both under- and oversegmentation [38,39]. In such cases, an extensive manual refinement, performed by expert radiologists, is required to correct the outcomes and achieve the desired standards. Therefore, the robustness and reliability of these automated tools towards various types of degenerated anatomies are crucial to integrate them into operational pipelines. Specifically, in the context of preoperative planning for TKA intervention, it is fundamental to guarantee a high degree of accuracy for the segmentation and 3D reconstruction, especially for specific regions, including tibial and femoral condyles. In these areas, often featured by the largest deformation and osteophytes, several contact points (Figure 4) are defined between the two bones and the PSI. Their potential mismatch might lead to the withdrawal of this innovative technique in favor of the traditional more invasive and time-consuming surgery [14]. This paper presented methodological improvements and a test in a real operational environment to address the limitations towards a true clinical translation of the previous related studies of our group [29,30]. A new data preparation pipeline, addressing a large dataset of 822 surgical cases, and featuring a patch-based method and a novel algorithm for the segmentation label generation, tackled both resolution and generalized oversegmentation problems previously observed. Patching the original CTs preserves their voxel size and retains fine-grained bone spatial details, whose identification is critical in this task. Moreover, accurate segmentation labels, tracing the bone boundaries even in the narrowest areas, boost the trained network in the localization of bone deformities and degraded shapes. Merging these approaches with the deployment of a novel CNN architecture tailored for bone boundary identification (CEL-UNet [30]) showed a strong improvement in segmentation and 3D reconstruction outcomes against our previous findings and traditional UNet models, leading to a drop in median RMSE values of 0.26 mm and 0.24 mm for the tibia and femur, respectively. These results were obtained on an independent set of 200 cases, considered adequate to assess the performances over the large variability in bone anatomies. In addition, sub-millimetric distance errors between crucial PSI contact areas on the reference and the reconstructed volumes as well as sub-angular alignment errors of the femoral and tibial cutting planes enhanced the applicability of this procedure in a real clinical context. Finally, the implementation of this automated technology within the *My Knee* department of MEDACTA proved its advantages over a standard manual segmentation process in terms of time saved for the overall process. Quality outcomes revealed that more than half of the processed cases already complied with the strict company requirement for PSI prototyping, reducing substantially the 3D reconstruction time to a median value of 13.1 s. For the remaining ones, the manual refinement process led to a drop in the elaboration times from four to 11 times, depending on the operator’s ability, compared to the original median value of 35 min.

### 4.2. Literature Comparison

Recent years have seen a rise in AI-based segmentation algorithms trying to replace the manual bone delineation process over different imaging acquisition techniques and anatomical regions. Deep CNNs were evaluated for skull surface segmentation in 20 CT scans to assist the surgical planning. Outcomes reported a sensitivity score of 0.92 and a 3D reconstruction error in the range of 1.5 mm [40]. To diagnose the severity of osteoarthritis in the shoulder joint, a UNet architecture was implemented to perform the humerus segmentation in shoulder CT images achieving a Dice coefficient of 0.946 [41]. However, the test set was composed of just 19 male subjects. Therefore, an actual validation or clinical translation of this study is controversial. A similar consideration can be provided for [42], where a fully automatic modified UNet model was proposed to detect and segment the tibia, femur, and patella, this time on knee osteoarthritic MRIs consisting of 160 2D slices for a single scan. Its performances were compared to traditional UNet and SegNet architectures. Results were tested over a 15-subject test set showing an overall Dice coefficient of 0.969 for their modified UNet, outperforming the other networks. Pelvic bone segmentation in 30 dual-energy CT scans was addressed by a traditional 3D-UNet achieving a Dice coefficient of about 0.958. In this study, the high memory demand of the 3D UNet architecture on the GPU was overcome by downsampling the input data to 128 × 128 × 128 voxels [43]. Finally, a 2D UNet designed to process the three anatomical planes in craniofacial CT was introduced to perform the mandibular bone segmentation. Outcomes reported a Dice index of 0.93 and reconstruction errors of 1.4 mm [44]. Despite some of the presented segmentation outcomes being numerically close to our achievements, the validation proposed in our study addresses a larger and more heterogeneous dataset including different degrees of several pathological conditions of the knee joint. In addition, the presented pipeline fully replaced manual operations in a relevant operational environment with promising results, proving once more the goodness of the procedure. Finally, the recorded median period to complete the whole pipeline is 13.1 s, considered compatible with the company’s requirements.

### 4.3. Technical Challenges and Work Limitations

The implementation of a robust and reliable AI-based automated algorithm for the segmentation and 3D reconstruction of the tibia and femur in the context of preoperative planning for TKA intervention poses several technical challenges. The perfect matching between the tibial and femoral PSI resection component to the bones during surgery strictly relies on the outcomes of this pipeline, which must perform even for severely degenerated and abnormal conditions. However, different sources of errors, at different steps of the procedure, might come together making this automated technology unfeasible for the current application. The first source of uncertainty is the scanning resolution, different for each acquisition machinery, and, in our dataset, with a median value of 0.43 mm. The bone segmentation introduces an additional error, a variable dependent on the goodness of the algorithm performances over each case. Moreover, the surface reconstruction step introduces greater uncertainties with larger slicing thicknesses. Finally, the surface smoothing further increases the difference between the true and generated patient bones. Nevertheless, an additional step should be considered in the overall pipeline. In particular, the PSI prototyping can decrease the matching accuracy due to manufacturing precision since modern 3D printers work with a 0.1 mm resolution. It was documented that a 1 mm uncertainty can lead to rotational disparities of approximately 2° in the coronal and sagittal planes when comparing the planned alignment to the one achieved during surgery [45,46]. For this reason, delivering 3D surfaces with sub-millimetric average reconstruction error is fundamental to ensure the most accurate match between bones and surgical instrumentation. Furthermore, bone structure, density, and size can exhibit variations across different ethnic groups, as demonstrated between Asian and Western populations [47]. Therefore, the predominance of specific groups in the dataset might bias the model towards unique bone morphologies and characteristics during the training phase. For this reason, a deeper study of the dataset composition could enhance the generalizability and results of a deep learning algorithm. To conclude, some important considerations should be provided to the computational feasibility of this procedure. The deployment of large CNN architectures, featuring hundreds of thousands of parameters, for large medical image segmentation tasks, can be successfully handled by dedicated machines characterized by considerable RAM and fast processors, as described in this work. However, general-purpose computers might lack the hardware required to perform such tasks, and extending these technologies to standard calculators can broaden their applications and usability. Reducing the computational overhead and memory allocation can overcome these limitations. Concerning the knee joint, performing separate segmentations of the tibia and femur, and further cropping the CT to focus on each bone alternatively, might overcome the memory allocation problem since smaller input volumes are provided to the network. Nevertheless, the overall computational time increases and this solution may not be generalizable for each domain. Accordingly, several studies showed how these challenges can be overcome through optimization techniques allowing network compression, including pruning and quantization [48,49,50]. Pruning reduces the number of parameters in the network, which directly decreases memory requirements during inference, while quantization reduces the memory footprint by representing values with fewer bits. In [49], the deep compression applied reduced the storage required by AlexNet by 35x, from 240MB to 6.9MB, and by VGG-16 by 49x, from 552MB to 11.3MB, both with no loss of accuracy. AlexNet was compressed by 51x, also in [50], while preserving the accuracy of the uncompressed network on ImageNet. These compression methods facilitate the use of complex neural networks in mobile applications where application size and download bandwidth are constrained. Their deployment in our work will make faster inference processes and a network most suitable for real-time applications, such as personalized preoperative planning performed by surgeons before the intervention.

## 5. Conclusions

The translation of AI-based tools into clinical practice recently emerged as a transformative avenue, enhancing traditional workflows in various medical domains. In this study, an AI-based pipeline built upon a multi-task CNN was investigated to replace the current semi-automatic segmentation and 3D reconstruction of the tibia and femur, in the context of preoperative planning for TKA intervention. Methodological progress, compared to the previous works of our group, significantly reduced the maximum and average bone reconstruction errors, with the latter being constantly below half a millimeter. The feasibility analyses of this approach for a PSI-based surgical plan revealed sub-millimetric distance errors and sub-angular alignment uncertainties in crucial surgical regions, such as the PSI contact areas and the two major cutting planes. Finally, the quality of the generated bone volumes was measured in a real operational environment, replacing manual operators in the processing of a set of surgical cases. A drastic time reduction to complete the whole procedure with comparable outcome accuracy was assessed proving the advantages and reliability of this approach compared to traditional semi-automatic methods. To conclude, the increasing collaboration between healthcare professionals and technology innovators is crucial to harnessing the full potential of these advancements, and a successful integration of AI into clinical practice is still an open challenge for the evolution of modern healthcare.

## Figures and Tables

**Table 1:** Dice, Jaccard, Recall, and Precision median and IQR scores for tibia (*above*) and femur (*below* comparing the performances of the three networks (CEL-, DCE-, and FOC-UNet). The * defines a statistically significant difference between the outcome distributions.

	Tibia			
	**Dice**	**Jaccard**	**Precision**	**Recall**
DCE-UNet	0.986 (0.982–0.989)	0.972 (0.965–0.978)	0.995 (0.992–0.997)	0.977 (0.970–0.985)
FOC-UNet	0.987 (0.983–0.989)	0.974 (0.966–0.979)	0.995 (0.992–0.998)	0.979 (0.971–0.985)
**CEL-UNet**	0.991 * (0.988–0.993)	0.982 * (0.977–0.987)	0.997 * (0.993–0.999)	0.986 * (0.980–0.994)
	**Femur**			
	**Dice**	**Jaccard**	**Precision**	**Recall**
DCE-UNet	0.987 (0.982–0.990)	0.974 (0.965–0.980)	0.994 (0.989–0.997)	0.980 (0.971–0.987)
FOC-UNet	0.988 (0.985–0.991)	0.976 (0.970–0.982)	0.995 (0.990–0.997)	0.982 (0.976–0.989)
**CEL-UNet**	0.992 * (0.990–0.994)	0.985 * (0.980–0.989)	0.996 * (0.993–0.998)	0.988 * (0.983–0.996)

**Table 2:** Time spent for each step in the pipeline, including preprocessing, segmentation, postprocessing, and 3D reconstruction of the tibia and femur. Tests were run on a 32-core CPU, and NVIDIA A100-PCIE GPU with 40 GB RAM.

	Preprocessing	Segmentation	Postprocessing	3D Reconstruction	Overall
Time (s)	1.1 (0.7–1.3)	4.4 (3.6–5.5)	3.8 (3.0–5.1)	4.2 (3.7–5.1)	13.1 (10.7–15.8)

**Table 3:** Angular alignment errors of the distal femur and proximal tibia cutting planes between the planning and predicted surfaces.

	Tibia		Femur	
	**Frontal**	**Sagittal**	**Frontal**	**Sagittal**
Error (°)	0.33 (0.24–0.46)	0.19 (0.13–0.35)	0.21 (0.13–0.38)	0.26 (0.12–0.36)

## Data Availability

The dataset presented in this article is not publicly available. Requests to access the dataset should be directed to pietro.cerveri@polimi.it.

## Abbreviations

The following abbreviations are used in this manuscript:

AI	Artificial intelligence
CEL	Combined Edge Loss
CNN	Convolutional Neural Network
CT	Computed Tomography
DCE	Distance Cross-Entropy
DICOM	Digital Imaging and Communications in Medicine
FOC	Focal
HU	Hounsfield Unit
IQR	Interquartile range
MRI	Magnetic Resonance Imaging
PSIs	Personalized Surgery Instruments
RMSE	Root Mean Squared Error
TKA	Total knee arthroplasty
TRL	Technology Readiness Level
